# The first complete genome sequence and pathogenicity characterization of fowl adenovirus serotype 2 with inclusion body hepatitis and hydropericardium in China

**DOI:** 10.3389/fvets.2022.951554

**Published:** 2022-08-15

**Authors:** Zimin Xie, Junqin Zhang, Minhua Sun, Qinghang Zeng, Yunzhen Huang, Jiawen Dong, Linlin Li, Shujian Huang, Ming Liao

**Affiliations:** ^1^Key Laboratory for Prevention and Control of Avian Influenza and Other Major Poultry Diseases, Institute of Animal Health, Guangdong Academy of Agricultural Sciences, Ministry of Agriculture and Rural Affairs, Scientific Observation and Experiment Station of Veterinary Drugs and Diagnostic Techniques of Guangdong Province, Ministry of Agriculture and Rural Affairs, Guangzhou, China; ^2^College of Life Science and Engineering, Foshan University, Foshan, China; ^3^Guangdong Laboratory for Lingnan Modern Agriculture, Guangdong, China; ^4^Guangdong Academy of Agricultural Sciences, Guangzhou, China

**Keywords:** fowl adenovirus serotype 2, complete genome, pathogenicity, weight gain, viral shedding

## Abstract

Since 2015, fowl adenovirus (FAdV) has been frequently reported worldwide, causing serious economic losses to the poultry industry. In this study, a FAdV-2, namely GX01, was isolated from liver samples of chickens with hepatitis and hydropericardium in Guangxi Province, China. The complete genome sequence of GX01 was determined about 43,663 base pairs (bp) with 53% G+C content. To our knowledge, this is the first FAdV-2 complete genome in China. There was a deleting fragment in ORF25 gene. Phylogenetic analysis based on the hexon loop-1 gene showed that GX01 is most closely related to FAdV-2 strain 685. Pathogenicity experiment of GX01 in 3-day-old and 10-day-old specific-pathogen-free chickens showed that although no mortality was observed within 21 days post infection (dpi), strain GX01 significantly inhibited weight gain of infected chickens. Moreover, FAdV-2 was still detectable in the anal swabs of infected chickens at 21 dpi. Necropsy analysis showed that the main lesions were observed in liver, heart, and spleen. Of note, hepatitis and hydropericardium were observed in the infected chickens. In addition, massive necrosis of lymphocyte was observed in spleen of infected 3-days-old chickens. We concluded that FAdV-2 strain GX01 is capable of causing hepatitis and hydropericardium, which will make serious impact on the growth of chickens. Our research lays a foundation to investigate the molecular epidemiology and etiology of FAdV.

## Introduction

Fowl adenoviruses (FAdVs), a non-enveloped, linear double-stranded DNA virus with a genome of 43–45 kbp in size, are common and harmful pathogens in chickens (1). FAdVs belongs to the family *Aviadenovirus*. According to the basis of phylogeny, genome organization and the lack of significant cross-neutralization (2, 3), it has currently been divided into five species (FAdV-A to FAdV-E) and 12 serotypes (FAdV-1 to FAdV-8a and FAdV-8b to FAdV-11) (4). FAdV-1 belongs to the species FAdV-A; FAdV-5 belongs to the species FAdV-B; FAdV-4 and FAdV-10 belong to the species FAdV-C; FAdV-2, 3, 9, and 11 belong to the species FAdV-D; FAdV-6, 7, 8a, and 8b belong to the species FAdV-E (1, 2, 5).

FAdVs are transmitted both vertically and horizontally, causing several diseases in chickens (6). These diseases include inclusion body hepatitis (IBH), hepatitis hydropericardium syndrome (HHS) and gizzard erosion (GE) (7–9). IBH is caused by all 12 serotypes (9–11). HHS is primarily related to FAdV-4 (12). And GE is mainly induced by FAdV-1, 8a, and 8b (13, 14). Since 2015, the epidemic trend of FAdVs has been gradually increasing worldwide, posing great threats to the poultry industry (6, 15). FAdV-2 has been detected in many countries, such as China (16, 17), South Africa (18, 19), Japan (2, 20), and Poland (21, 22). It mainly infected 3–5 weeks broiler, resulting in severe IBH with mortality rates ranging from 0–17% (16, 20, 23). Up till now, only one complete genome sequence of defined FAdV-2 is available in GenBank. Moreover, the pathogenicity of FAdV-2 is still unclear, especially the impact on weight and viral shedding (2, 24, 25).

In our study, we successfully isolated a FAdV-2 strain GX01 from commercial broiler with hepatitis and hydropericardium. The complete genome sequence of GX01 was determined and characterized. The pathogenicity of GX01 was evaluated in 3-day-old and 10-days-old Specific Pathogen Free (SPF) chickens. To our knowledge, this work reports the first FAdV-2 complete genome sequence in China. The pathogenicity experiment showed that FAdV-2 strain GX01 is capable of causing hepatitis and hydropericardium. Moreover, GX01 significantly inhibited weight gain and caused viral shedding for a long period of time in the infected chickens. We concluded that the isolated GX01 may have serious impact on the production of the poultry industry, especially the broiler industry.

## Materials and methods

### Sample collection and virus isolation

In July 2020, the liver samples were collected from 20-days-old commercial broiler with hepatitis and hydropericardium in Guangxi Province, China. Total RNA and DNA were extracted using Tianlong Nucleic Acid Extraction & Purification Kit T180H (Tianlong Technology Co., Ltd., China). The extracted DNA was subjected to polymerase chain reaction (PCR) amplification to detect FAdV (26). Other pathogens include avian influenza virus (AIV), Newcastle disease virus (NDV), infectious bursal disease virus (IBDV), infectious laryngotracheitisand virus (ILTV), chicken infectious anemia virus (CIAV), avian leukosis virus (ALV), Marek's disease virus (MDV), egg drop syndrome virus (EDSV) and avian reovirus (ARV) were detected by PCR or reverse transcription-polymerase chain reaction (RT-PCR) assays. The positive PCR product was sequenced. And the obtained nucleotide sequence was blasted in NCBI. The primers used for detecting pathogens here were shown in Supplementary Table 1. And the primers were synthesized by Sangon Biotech (Guangzhou, China). The liver sample was homogenized in phosphate buffered saline (PBS) to obtain a 10% tissue suspension. After three freeze-thaw cycles, the suspensions were centrifuged at 12,000 × *g* at 4°C for 10 min. And then the suspensions were filtered through a 0.22-μm pore-size sterile filter (Millipore, Bedford, MA, United States). The filtered solution was inoculated into leghorn male hepatocellular (LMH) cells and propagated for three passages (27, 28). The infected LMH cells and the mock LMH cells were sent to Guangzhou Sevier Biotechnology Co., Ltd., China for transmission electron microscopy (TEM) analysis.

### TCID_50_ and growth curve assay

The 50% tissue culture infective dose (TCID_50_) assay was performed as previously described (29). Viral cytopathic effect (CPE) was observed for approximately 5 days. The virus titer was calculated as TCID_50_ according to the Reed-Muench method (30). The virus growth kinetics were determined as previously described with a few modifications (31). Briefly, the supernatant and infected LMH cells were harvested at 0, 12, 24, 36, 48, 60, 72, 84, and 96 h post infection (hpi), respectively. After three freeze-thaw cycles, the virus titers of each time point were determined by TCID_50_ assay.

### Determination of complete genome

Based on published sequences of FAdVs, a set of primers were designed to determine the complete genome sequence of GX01 (Supplementary Table 2). And the primers were synthesized by Sangon Biotech (Guangzhou, China). The fragments of GX01 were amplified by PCR assay. The amplified fragments were determined by sequencing (Shanghai Sangon Biotechnology Co., Ltd., China). Finally, all sequences were assembled using the SeqMan of the DNASTAR software package (version 7.1, Madison, WI, United States). The open reading frames (ORFs) of complete genome sequence were annotated using the Snapgene software (version 2.3.2, United States).

### Phylogenetic analysis

Based on the sequences of GX01 complete genome and hexon loop-1 gene and amino acid sequences of hexon, fiber, and DNA polymerase gene, the homology analysis of GX01 was carried out with other FAdV-D strains reference sequences using the MegAlign of the DNASTAR package. Moreover, Phylogenetic trees were constructed based on the sequences of DNA polymerase amino acid and hexon loop-1 gene, respectively, by the maximum likelihood method in MEGA 7.0 software (32). Bootstrap values were determined based on the original data from 1,000 replicates.

### Animal experiment and ethics statement

Twenty 3-days-old and 20 10-days-old SPF leghorn chickens (Guangdong Dahuanong Animal Health Products Co., Ltd., China) were raised in separated negative-pressure isolators and randomly divided into two groups, respectively. Chickens in the challenge groups were inoculated intravenously with dose of 0.2 ml (1.0 × 10^7^ TCID_50_/ml) virus. The control groups were inoculated intravenously with an equal volume of DMEM/F12 basic medium (Gibco, Australia). All chickens were monitored daily for clinical signs. The anal swabs of all chickens were collected from 0 to 21 dpi. The weight of all chickens was recorded weekly. At 21 dpi, all chickens were humanely euthanized. The liver, heart, spleen, and kidney were collected. And the weight of spleen was recorded. The spleen indexes were calculated by the spleen (milligram, mg) / body weight (gram, g). For histopathological examinations, samples were fixed in 4% paraformaldehyde. The remaining samples were stored at−80°C. The animal experiment protocol used in this study was approved by and performed under the guidance of the Committee on the Ethics of Animal Experiments of Institute of Animal Health, Guangdong Academy of Agricultural Sciences Experimental Animal Welfare Ethics Committee on 8 November, 2021 (Approve ID: SPF2021027). All efforts were made to minimize animal suffering.

### Real-time quantitative PCR

Total DNA of anal swabs and tissue samples were extracted. Quantitative real-time PCR analysis was carried out using SYBR Green master mix (Roche, United states). Absolute quantitative real-time PCR was conducted as described previously with small modifications (33). Briefly, 200 microliter (μl) was taken from anal swab to obtain DNA in 60 μl volume. 200 mg was taken from tissue sample to obtain DNA in 60 μl volume. And then 1 μl was taken to conduct real-time PCR. Primers were designed according to the conserved 52K gene of FAdVs (Supplementary Table 3) (33). A plasmid containing the 52K gene of FAdV-2 strain was used to construct standard curve in each reaction.

## Results

### Virus isolation and identification

**Figure 1:**
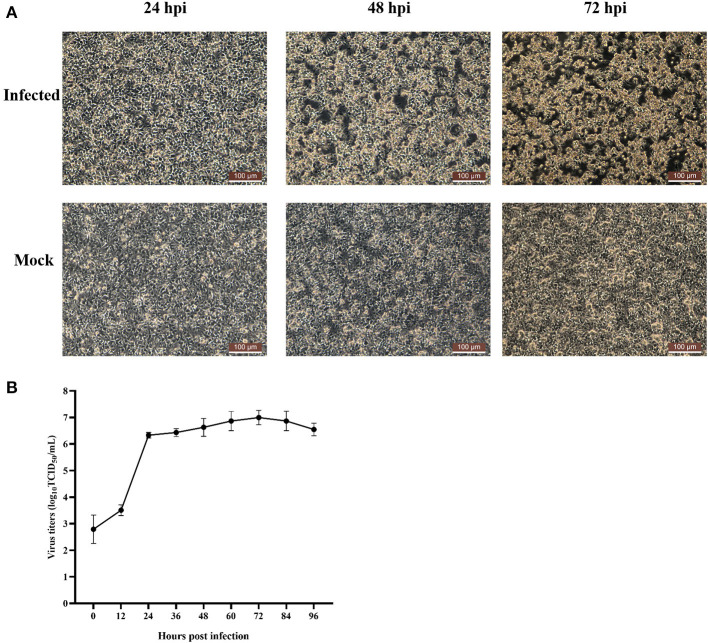
Cytopathic effect of FAdV-2 strain GX01 infected LMH cells and one-step growth curve. **(A)** Cytopathic effect of FAdV-2 strain GX01 infected LMH cells and non-infected LMH cells (Mock) at 24, 48, and 72 hpi. Scale bar = 100 μm. **(B)** One-step growth curve of FAdV-2 strain GX01 in LMH cells.

**Figure 2:**
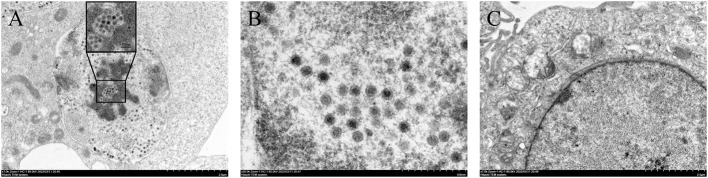
Transmission electron micrographs of LMH cells. **(A)** and **(B)** are transmission electron micrographs of viral particles; **(C)** showing transmission electron micrographs of normal LMH cells.

In July 2020, chickens on a chicken farm in Guangxi Province were suffering from severe hepatitis and mild hydropericardium. The liver samples were tested positive for FAdV but tested negative for AIV, NDV, IBDV, ILTV, CIAV, ALV, MDV, EDSV, and ARV. After inoculating the filtered tissue supernatant in LMH cells, the infected LMH cells were shedding, round-shaped and strongly refracted at 48 dpi to 72 dpi (Figure 1A). According to the growth curve of strain GX01, the virus titer increased dramatically from 12 hpi to 24 hpi, and then it reached a plateau at 24 hpi and peaked at 72 hpi, approximately 1.0 × 10^7^ TCID_50_/ml (Figure 1B). Moreover, the virus particles were observed by TEM. It displayed a spherical shape and latticed distribution (Figure 2). These results indicated that the virus was successfully isolated. The isolated virus was designated as GX01.

### Complete genome analysis

**Figure 3:**
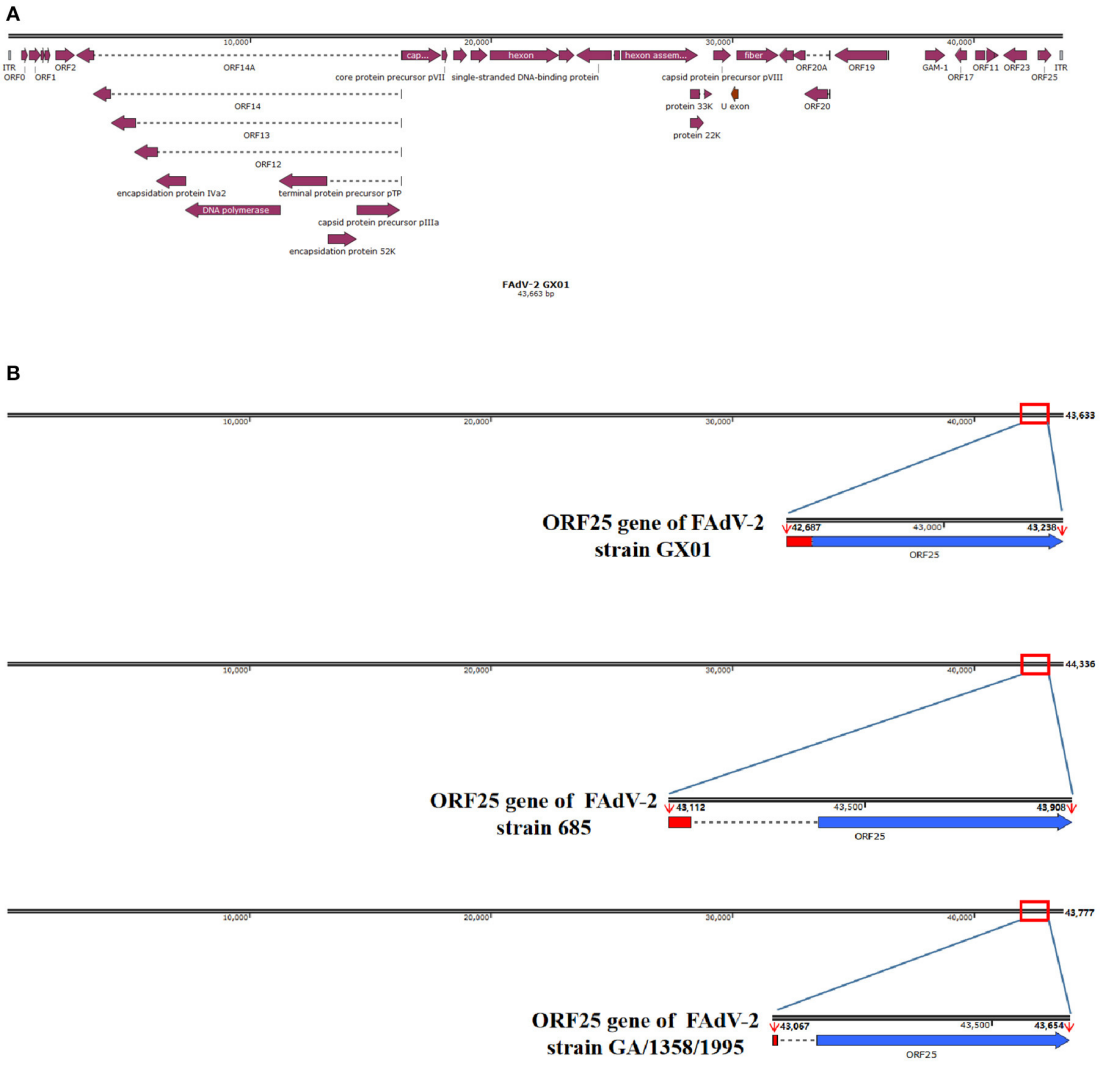
Schematic representation of FAdV-2 strain GX01 and ORF25 gene. **(A)** The complete genome schematic representation of FAdV-2 stain GX01; **(B)** Comparison of ORF25 gene of FAdV-2 strain GX01 with ORF25 gene of other FAdV-2 strains (red region: the first coding sequence of ORF25; blue region: the second coding sequence of ORF25; dotted line: non-coding sequence).

To obtain the complete genome sequence of strain GX01, all the sequences were assembled together. Finally, we obtained a 43,663 bp viral genome sequence of strain GX01, with 53% G + C content. And the complete genome sequence contained 36 ORFs (Figure 3A). The complete genome sequence is deposited in GenBank under the accession number ON014843. In addition, there was a deleting fragment in ORF25 gene (Figure 3B). The two coding sequences (CDS) of GX01 ORF25 gene are contiguous. The ORF25 gene of other FAdV-2 strains has a non-coding sequence in the middle of the CDS at both ends.

### Phylogenetic analysis and sequence comparisons of strain GX01

**Figure 4:**
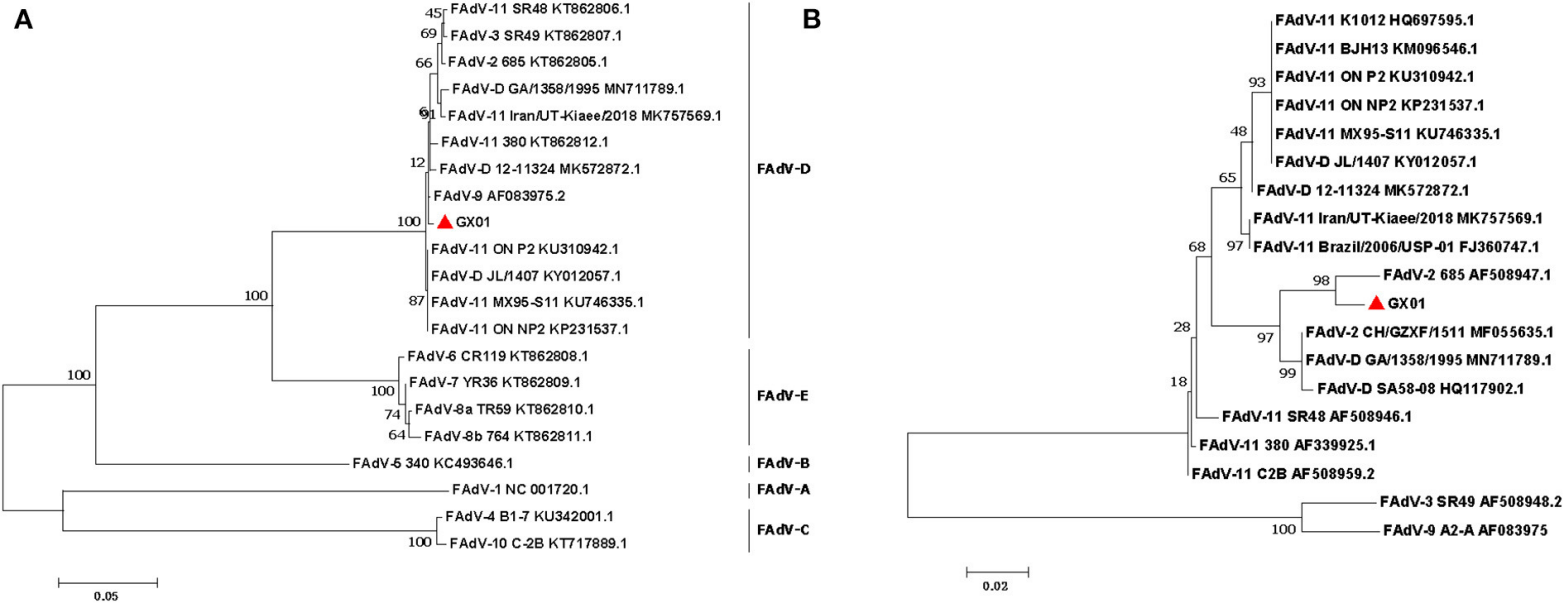
Phylogenetic analysis of strain GX01. The phylogenetic trees were constructed by the maximum likelihood method in MEGA 7.0. Bootstrap majority consensus values based on 1,000 replicates are indicated at each branch point as a percentage. **(A)** Phylogenetic tree based on amino acid sequence of DAN polymerase. **(B)** Phylogenetic tree based on the nucleotides of hexon loop-1 gene.

According to the species demarcation criteria of the International Committee on Taxonomy of Viruses (ICTV), the species designation of FAdVs depends on the distance matrix analysis of the DNA polymerase amino acid sequence. Phylogenetic tree based on amino acid sequence of DNA polymerase showed that GX01 was clustered within the species FAdV-D (Figure 4A). In addition, the hexon loop-1 gene is recognized as a gene that distinguishes serotypes of FAdVs (4). Phylogenetic tree based on hexon loop-1 gene showed that strain GX01 was most closely related to FAdV-2 strain 685 (Figure 4B).

Pairwise comparisons were performed to determine the sequence identities. The results showed that GX01 shared of 90.5–97.9% identify in the complete genome sequence with other FAdV-D strains (Table 1). The homology analysis based on hexon loop-1 gene showed that GX01 shared 97.7% identity with FAdV-2 strain 685. The sequence identities at the amino acid level were 89.5–98.6% (hexon), 78.1–95.9% (fiber), and 90.1–99.3% (DNA polymerase) between GX01 and other FAdV-D strains.

**Table 1:** Comparison of complete genome sequences, hexon loop-1 gene and amino acid sequences of the three ORFs of GX01 with other FAdV-D strains.

**Species**	**Virus**	**GenBank accession no**.	**Complete genome sequence in legth (nt)**	**Sequence identity compare with GX01 (%)**				
				**Genome (nt)**	**Hexon loop-1 (nt)**	**Hexon** **(aa)**	**Fiber (aa)**	**DNA polymerase** **(aa)**
FAdV-D	FAdV-2 strain 685	KT862805.1	44,336	96.1	97.7	98.6	94.9	99.2
	FAdV-2 strain CH/GZXF/1511	MF055635	-	-	96.8	-	-	-
	FAdV-2 strain ATCC ATCC VR-827	AF339915.1	-	-	96.7	-	-	-
	FAdV-3 strain SR49	KT862807.1	43,337	90.5	78.6	90.0	78.1	98.9
	FAdV-9 strain A2-A	AF083975.2	45,063	93.3	79.1	89.5	84.7	90.1
	FAdV-11 strain 380	KT862812.1	43,347	97.6	94.6	97.6	88.7	99.0
	FAdV-11 strain SR48	KT862806.1	43,632	97.9	94.1	97.5	95.9	99.3
	FAdV-11 strain MX95-S11	KU746335.1	44,326	96.4	95.7	98.6	95.7	99.1
Unassigned	FAdV-D strain GA/1358/1995	MN711789.1	44,079	96.2	96.8	97.9	95.7	98.6
	FAdV-D strain 12-11324	MK572872.1	43,405	97.5	95.0	98.6	94.7	99.1
	FAdV-D strain JL/1407	KY012057.1	44,054	97.1	95.7	98.6	95.6	99.1

### Pathogenicity assessment

**Figure 5:**
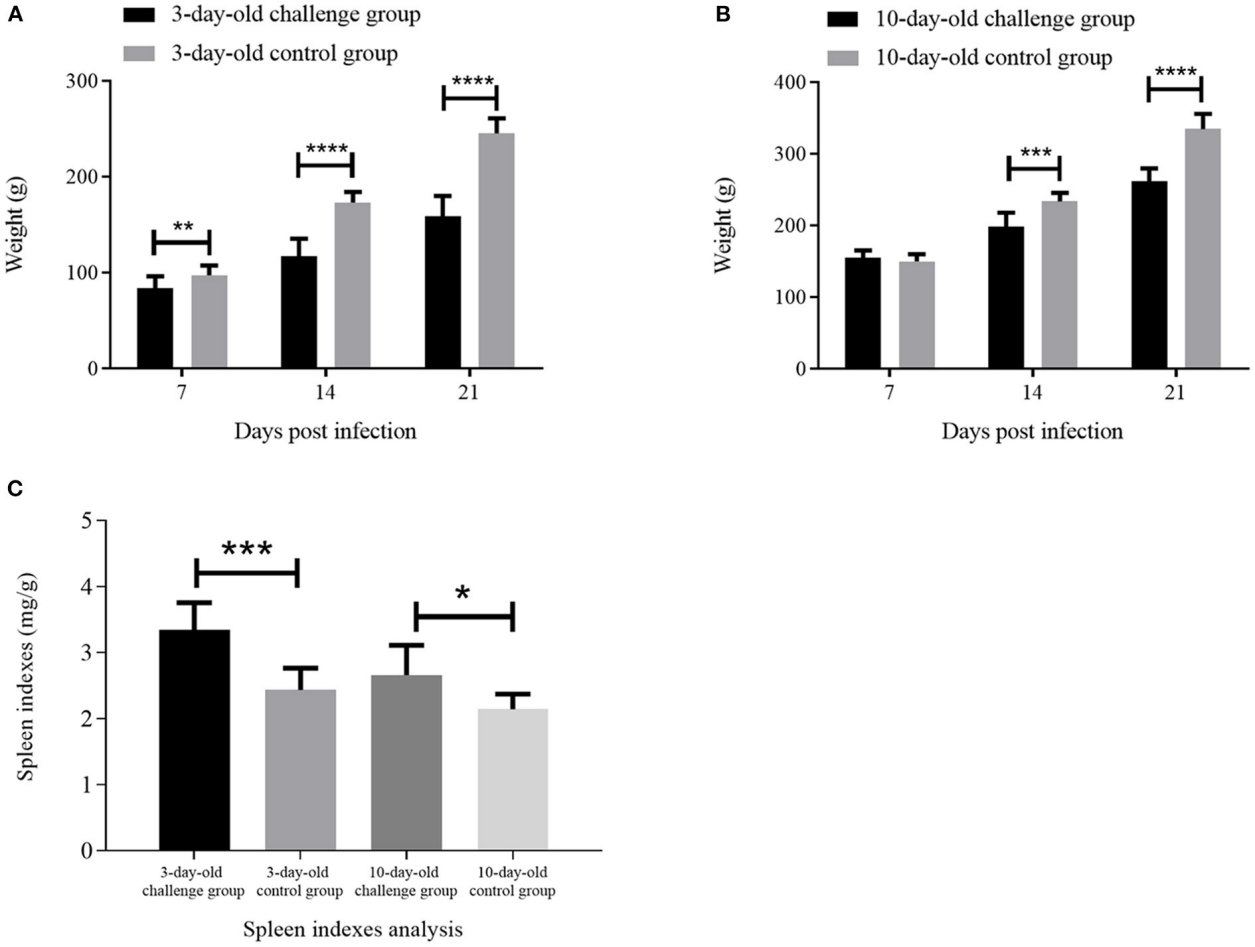
The weight analysis and spleen indexes analysis. **(A)** and **(B)** are the weight analysis of the challenge groups and control groups at 7, 14, and 21 days post infection (dpi); **(C)** is the spleen indexes analysis of challenge groups and control groups at 21 dpi. * represents *p* < 0.05; ** represents *p* < 0.01; *** represents *p* < 0.001; **** represents *p* < 0.0001.

In order to assess the pathogenicity of GX01 to chickens, we used GX01 to challenge chickens. All of chickens in the challenge groups showed clinical signs of depression and anorexia at 1 to 3 dpi. The chickens in the control groups did not show any clinical signs. Moreover, although none of the chickens died through the study, the weight gain of infected 3-days-old chickens was significantly inhibited than that of the control group at 7, 14, and 21 dpi. The average weight of control chickens reached 245 g at 21 days, while the average weight of infected chickens was only 159 g (Figure 5A). The weight gain of infected 10-day-old chickens was significantly inhibited than that of the control group at 14 and 21 dpi. The average weight of control chickens reached 334 g at 21 days, while the average weight of infected chickens was only 261 g (Figure 5B).

**Figure 6:**
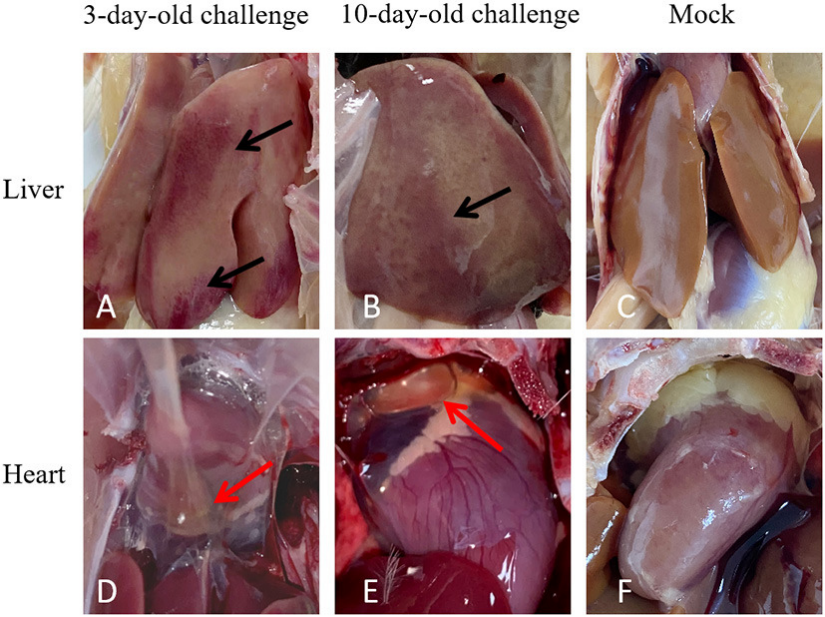
Pathogenicity analysis of FAdV-2 strain GX01. Clinical signs were observed in the challenge and negative control groups at 21 days post infection: livers enlargement and hemorrhage (Black arrow) and hearts mild hydropericardium (Red arrow). **(A,B)** Abnormal liver in 3-day-old challenge group and 10-day-old challenge group respectively. **(C)** Normal liver in mock group. **(D,E)** Abnormal heart in 3-day-old challenge group and 10-day-old challenge group, respectively. **(F)** Normal heart in mock group.

**Figure 7:**
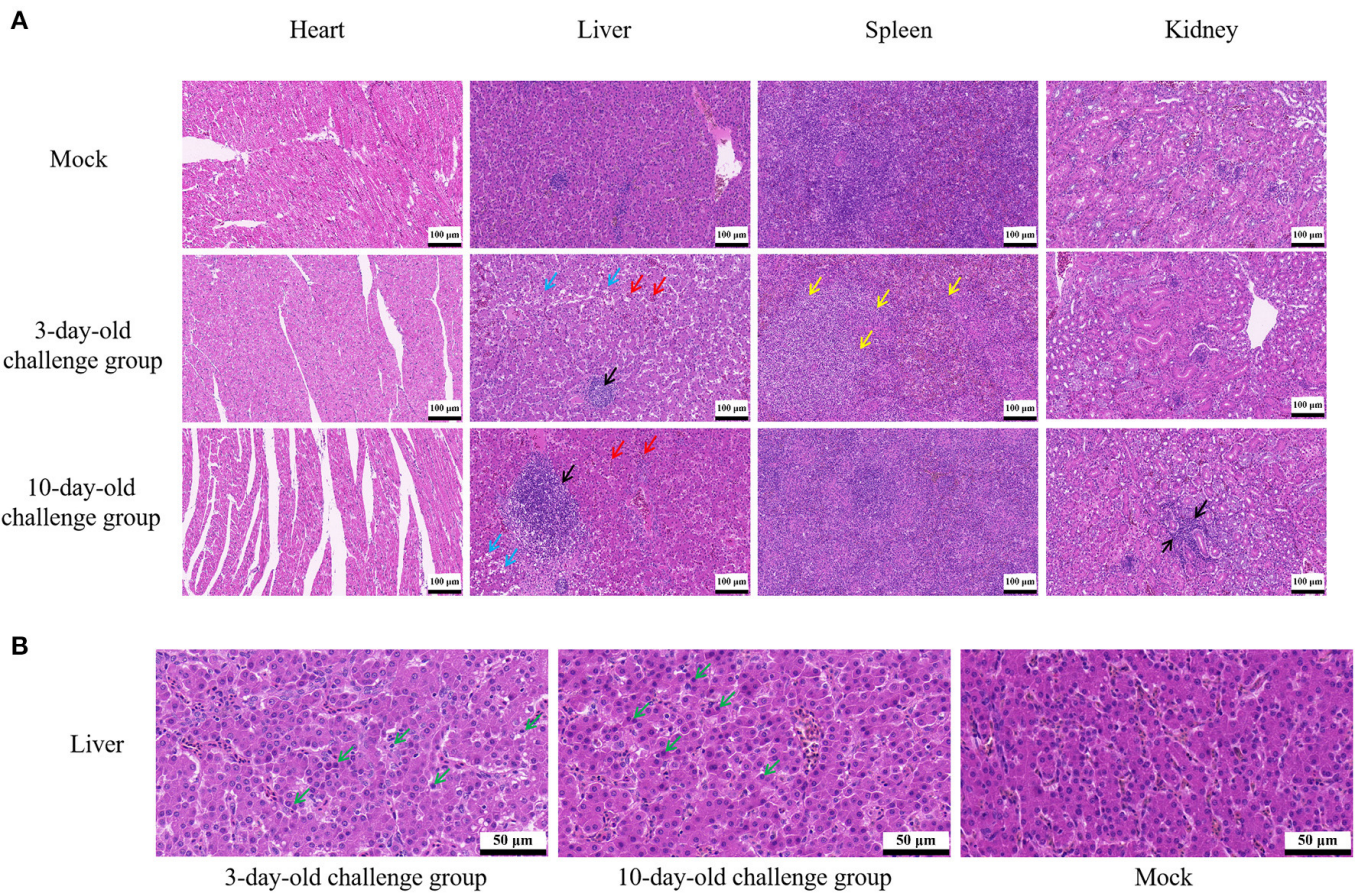
Histopathological analysis of FAdV-2 strain GX01. **(A)** Histopathological analysis showed massive infiltration of inflammatory cells was observed in liver and kidney of infected chickens (black arrow); Congested in liver sinuses (red arrow); Hepatocyte vacuolar degeneration with small vacuoles visible in the cytoplasm (blue arrow); Massive lymphocyte necrosis in red-white pith of spleen (yellow arrow). **(B)** Typically inclusion body was observed in liver (green arrow).

At 21 dpi, necropsy results showed that livers of challenge groups exhibited obvious petechial hemorrhage (Figures 6A,B). And the pericardium showed yellowish effusion (Figures 6D,E). In addition, the spleen indexes showed that the spleens in challenge groups were significantly larger than that of the control groups (Figure 5C). Histological examination showed that massive infiltration of inflammatory cells was observed in liver and kidney of infected chickens (Figure 7A). Typically IBH was observed in livers of 3-days-old challenge and 10-days-old challenge groups (Figure 7B). Lesions in the spleen were characterized as massive lymphocyte necrosis in red-white pith. Absolute quantification of FAdV-2 in the organs of the euthanized chickens showed that the FAdV-2 was detectable in the heart, liver, spleen, and kidneys. The viral loads were still remained 60 to 4.67 × 10^2^ copies (Supplementary Figure 1). No FAdV-2 was detected in the negative control groups.

### Viral shedding analysis

**Figure 8:**
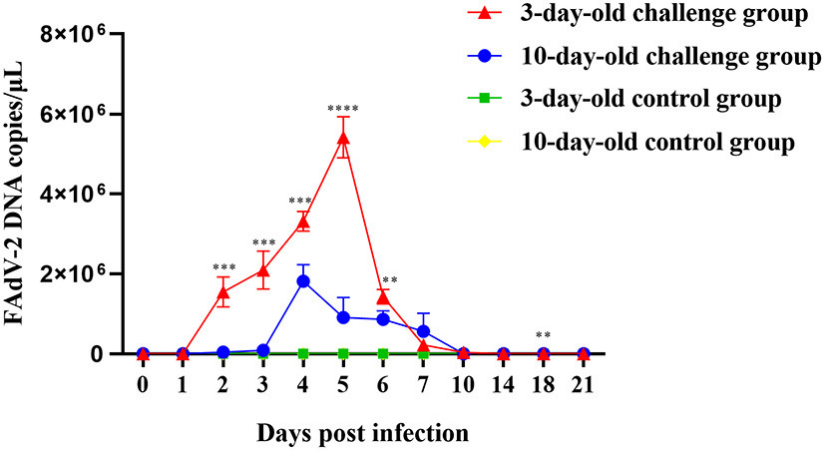
Viral shedding of FAdV-2 strain GX01. The viral shedding of challenge and negative control groups at 0, 1, 2, 3, 4, 5, 6, 7, 10, 14, 18, and 21 days post infection (Asterisk represents the significant difference in virus shedding between the 3-day-old challenge group and the 10-day-old challenge group on the same day).

To determine viral shedding of GX01 in the 3-days-old and 10-days-old SPF chickens, we collected the anal swabs of all chickens from 0 to 21dpi. The virus genome was detected in anal swabs by quantitative real-time PCR assays (Figure 8). At 1 dpi, FAdV-2 in infected 3-days-old and 10-days-old groups has been detected about 1.26 × 10^2^ copies and 1.93 × 10^2^ copies, respectively. The viral shedding of infected 3-days-old chickens increased sharply at 3dpi and peaked at 5dpi (5.42 × 10^6^ copies), then it dramatically declined. The viral shedding of infected 10-days-old chickens increased sharply at 3dpi and peaked at 4dpi (1.82 × 10^6^ copies), then it declined slowly. At 21 dpi, FAdV-2 in infected 3-days-old and 10-days-old groups was still detectable approximately 2.64 × 10^2^ copies and 1.26 × 10^2^ copies, respectively. No FAdV-2 was detected in the negative control chickens throughout the experiment.

## Discussion

In recent years, FAdVs have been frequently reported worldwide, causing huge economic loss to the poultry industry (34). Co-infection of multiple serotypes was observed in different regions, such as FAdV-2 and 8b in South Africa (18, 19), FAdV-2, 8a, 8b, and 11 in Asia, Europe and North America (2, 15, 21, 35–37). In China, FAdVs are transmitted both vertically and horizontally. The prevalence is dominated by serotype 4, 8a, 8b and 11 (23). Co-infection has also been reported in southern China (23). In this work, we successfully isolated a FAdV-2 strain GX01 and obtained the complete genome sequence of the virus. Moreover, the pathogenicity of FAdV-2 strain GX01 was deeply evaluated in 3-old-days and 10-old-day SPF chickens.

Up till now, only two complete genome sequences of FAdV-2 are documented in GenBank. They are FAdV-2 strain 685 and FAdV-2 strain SR48. FAdV-2 strain 685 was isolated from United Kingdom. FAdV-2 strain SR48 has already been confirmed to be FAdV-11 (2). Besides, our study confirmed the unassigned species FAdV-D strain GA/1358/1995 to be a FAdV-2, based on phylogenetic analysis of hexon loop-1 gene. The hexon gene of FAdVs was related to the classification of serotypes, especially the hexon loop-1 gene (4, 26, 35). This gene is sufficiently variable to ensure species identification and additional differentiation of the currently recognized 12 serotypes (38–40). Moreover, the ORFs of GX01 were almost identical to the other reported FAdV-2 strains by alignment with reference sequences, suggesting that the genome sequence of FAdV-2 was relatively conserved. However, there are some differences in ORF25 gene of GX01. The ORF25 gene of other strains in species FAdV-D is composed of two discontinuous CDS (5). In our study, the two CDS of GX01 ORF25 gene were contiguous, indicating that some non-coding sequences of GX01 were deleted in natural evolution. Moreover, the sequence of the first CDS of GX01 ORF25 gene was quite different from the other known FAdV-2 strains. To date, the function of FAdVs ORF25 gene is still unclear (25, 41, 42). It may be associated with evolution and mutation of the virus.

The pathogenicity of FAdV-2 is not well–understood, especially with regard to the effects on weight gain and viral shedding. In our study, although none of the chickens died throughout the experiment, we found the weight gain of infected chickens was significantly inhibited than that of control groups. It means that the growth of chickens is affected by FAdV-2 strain GX01, which will cause significant economic losses to poultry industry. Moreover, the viral shedding increased sharply, indicating strain GX01 has a strong ability to proliferate in chickens. Furthermore, FAdV-2 was still detectable in anal swabs at 21 dpi, indicating viral shedding lasted for a long period of time after infection, which will produce significant cross-infection in production and endanger healthy flocks.

FAdV-2 has only been reported to cause IBH (16). HHS was related with FAdV-4 and 11 (12, 43). Interestingly, in addition to severe hepatitis, hydropericardium was also observed in the infected chickens in our study. Thus, GX01 was able to cause HHS in both clinical and experimental cases. To our knowledge, this is the first report in which hydropericardium were associated with the FAdV-2. The volume of hydropericardium caused by FAdV-4 can reach as much as 15 ml, while the volume of that caused by FAdV-2 strain GX01 and FAdV-11 was 1 to 2 ml (43, 44). We suggested that the pathogenicity of FAdV-2 strain GX01 may be diverse.

In recent years, FAdV-4 has been proven to have immunosuppressive potential, which caused structural and functional damage of immune organs *via* apoptosis along with induction of severe inflammatory responses (45–47). Other serotypes of FAdVs have also been reported to cause immune system damage in chickens, such as FAdV-8b, and FAdV-11 (48–50). In our study, not only the spleen indexes of challenge groups were significantly higher than that of the control groups, but also massive necrosis of lymphocyte was observed in spleen of infected 3-days-old chickens. These result revealed that GX01 may also be an immunosuppressive pathogen. The immunosuppression potential of FAdV-2 is required for further investigation.

## Conclusions

In our study, a FAdV-2 strain GX01 was successfully isolated from commercial broiler with HHS in Guangxi Province, China. The first complete genome of FAdV-2 in China was determined and characterized, which not only increased the knowledge of the molecular characteristics, but also enriched the understanding of FAdV-2 diversity. Moreover, the Pathogenicity of GX01 in SPF chickens showed that GX01 significantly inhibited weight gain in infected chickens and caused viral shedding lasted for at least 21 dpi. Furthermore, FAdV-2 strain GX01 is capable of causing HHS. We concluded that this virus may become a severe threat to poultry industry. Therefore, further studies of pathogenic mechanism and vaccine development of FAdV-2 are needed to obtain more insights for the prevention and control of this disease.

## Data availability statement

The original contributions presented in the study are included in the article/Supplementary material, further inquiries can be directed to the corresponding author.

## Ethics statement

The animal study was reviewed and approved by the Committee on the Ethics of Animal Experiments of Institute of Animal Health, Guangdong Academy of Agricultural Sciences Experimental Animal Welfare Ethics Committee on 8 November, 2021 (Approve ID: SPF2021027).

## Author contributions

ZX and JZ designed this study and critically revised the manuscript and performed the experiments, data analysis, and drafted the manuscript. ZX, MS, QZ, YH, JD, and LL participated in sample collection, virus isolation, and animal experiment. SH and ML participated in the coordination and manuscript revision. All authors read and approved the final manuscript.

## Funding

This work was funded by Special Fund for Scientific Innovation Strategy-Construction of High Level Academy of Agriculture Science (202110TD, R2020PY-JX014, R2020QD-049, and R2020PY-JC001), Guangzhou Basic and Applied Basic Research Foundation (202102021162), Guangdong Laboratory for Lingnan Modern Agriculture Project (NZ2021015), Guangdong Basic and Applied Basic Research Foundation (2018A0303130003), Scientific Research Plan of Guangzhou Science and Technology Project (201904010379), Collaborative Innovation Center of GDAAS (XTXM202202), the Science and Technology Planning Project of Jiangmen (2020030103310009038), Guangdong Basic and Applied Basic Research Foundation (2019B1515210008), Key Research and Development Program of Guangdong Province (2020B0202090004), Yunnan Liao Ming Expert Workstation (202105AF150077), Science and Technology Program of Guangdong Province (2021B1212030015), and Zengcheng District Entrepreneurial Leading Team Project (2021).

## Conflict of interest

The authors declare that the research was conducted in the absence of any commercial or financial relationships that could be construed as a potential conflict of interest.

## Publisher's note

All claims expressed in this article are solely those of the authors and do not necessarily represent those of their affiliated organizations, or those of the publisher, the editors and the reviewers. Any product that may be evaluated in this article, or claim that may be made by its manufacturer, is not guaranteed or endorsed by the publisher.
