# Supplementary material for: The first complete genome sequence and pathogenicity characterization of fowl adenovirus serotype 2 with inclusion body hepatitis and hydropericardium in China
Source: Front Vet Sci. 2022 Aug 15;9:951554. doi: 10.3389/fvets.2022.951554 (PMC9443503; doi:10.3389/fvets.2022.951554)
Supplement: Supplementary file 1 [file Table_1.DOCX]

TABLE 1. Primers of PCR assay

| Primers | Sequence (5’ to 3’) | Length, (bp) |
| --- | --- | --- |
| FAdV-F | CAARTTCAGRCAGACGGT | 897 |
| FAdV-R | TAGTGATGMCGSGACATCAT |  |
| AIV-F | TTCTAACCGAGGTCGAAAC | 229 |
| AIV-R | AAGCGTCTACGCTGCAGTCC |  |
| NDV-F | ATGGGCYCCAGAYCTTCTAC | 535 |
| NDV-R | CTGCCACTGCTAGTTGTGATAATCC |  |
| IBDV-F | AGCCTTCTGATGCCAACAAC | 365 |
| IBDV-R | ATCTGTCAGTTCACTCAGGC |  |
| ILTV-F | GTAACTGACTACGCATC | 1112 |
| ILTV-R | TTAGCACAGACACGCA |  |
| IAV-F | GACTGTAAGATGGCAAGACGAGCTC | 675 |
| IAV-R | GGCTGAAGGATCCCTCATTC |  |
| ALV-F | GGATGAGGTGACTAAGAAAG | 545 |
| ALV-R | CGAACCAAAGGTAACACACG |  |
| MDV-F | TGTTCGGGATCCTCGGTAAGA | 583(763) |
| MDV-R | AGTTGGCTTGTCATGAGCCAG |  |
| EDSV-F | AGGTGTCTGATATTGGAGTG | 110 |
| EDSV-R | TGGATGAAACGCTTTATAAG |  |
| ARV-F | GGTGCGACTGCTGTATTTGGTAAC | 532 |
| ARV-R | AATGGAACGATAGCGTGTGGG |  |

TABLE 2. The fragments primer of FAdV-2 strain GX01

| Primers | Sequence (5’ to 3’) | Location of primers | Length, (bp) |
| --- | --- | --- | --- |
| GX01-F1 | CATCATCTATATATACCTACATGAAT | 1-26 | 1417 |
| GX01-R1 | CCTCGTTATCTGCCTCGAGA | 1398-1417 |  |
| GX01-F2 | AAGAGCGATCGGGTTTAGAC | 1241-1260 | 1546 |
| GX01-R2 | TTGGCAAACACGAAACACGC | 2767-2786 |  |
| GX01-F3 | GCTTGTCGCACGTTAGTCAA | 2573-2592 | 1417 |
| GX01-R3 | CCGTTCCAGTCCTCCTTTA | 3974-3992 |  |
| GX01-F4 | GGACTGACAAAAGAGCGAGA | 3861-3880 | 1533 |
| GX01-R4 | CAATACCTGAGGCAGATACG | 5375-5394 |  |
| GX01-F5 | CAGGAGTAACACGGGGGATC | 5285-5304 | 1506 |
| GX01-R5 | ATGACGTACGAGGAAGCAAC | 6771-6790 |  |
| GX01-F6 | CAGAGTTTATCCATGCACTCG | 6666-6686 | 1529 |
| GX01-R6 | CTGCTTCGTATTGGGATGGT | 8175-8194 |  |
| GX01-F7 | TCTCCTTCATGCGCCGATA | 8031-8049 | 1560 |
| GX01-R7 | TGCACGGCGACTTTAACAT | 9572-9590 |  |
| GX01-F8 | GGTTCCCCTTGATGTCGTT | 9411-9429 | 1511 |
| GX01-R8 | AAGACTGCCCGTCTTCAAAC | 10902-10921 |  |
| GX01-F9 | GTTCTGGTCTTGGGTGACCA | 10792-10811 | 2029 |
| GX01-R9 | ACTGCTCGTACTCCGTCAA | 12802-12820 |  |
| GX01-F10 | GGAGGCGATTCTGTCCATAA | 12708-12727 | 1451 |
| GX01-R10 | CGCGATCTTAGCGTATTTCAT | 14138-14158 |  |
| GX01-F11 | AATACCATGCTCGCCCAACG | 13982-14001 | 1605 |
| GX01-R11 | CGCCAGTCTCGGTCCATCAC | 15567-15586 |  |
| GX01-F12 | AGGATTCCTTCGGACGTTTC | 15423-15442 | 1796 |
| GX01-R12 | GCGTTTCGCTATCCCTAACA | 17199-17218 |  |
| GX01-F13 | TCGGCGTTAAATTCGACACG | 17043-17062 | 1898 |
| GX01-R13 | GCGGCAGCGATGATGGGTAT | 18921-18940 |  |
| GX01-F14 | AAGGAGAAACGGTGTTAGCG | 18846-18865 | 1573 |
| GX01-R14 | GCCCCGTGATGACTGTCTTG | 20399-20418 |  |
| GX01-F15 | GTGGGCGACAGTTGGGTTCT | 20241-20260 | 1786 |
| GX01-R15 | AGGTGCCGTCAGAGTAGGGA | 22007-22026 |  |
| GX01-F16 | ATGCTCAGAAACGCCACTAA | 21789-21808 | 1983 |
| GX01-R16 | TCGCAAGACCTCCGATAAAA | 23751-23770 |  |
| GX01-F17 | TCACCTGTTTCAACGGGTTT | 23573-23592 | 1998 |
| GX01-R17 | CGGATTCGCGTCTAGGAGGT | 25551-25570 |  |
| GX01-F18 | AATCTCGTCGTCAATACCGC | 25435-25454 | 1970 |
| GX01-R18 | ACTATAGCTTTGGACACCTC | 27385-27404 |  |
| GX01-F19 | AGCCATAGACGACACCACAA | 27300-27319 | 1882 |
| GX01-R19 | ATGAAAGGGATCTCCACAGG | 29162-29181 |  |
| GX01-F20 | TTGAATCTGCTCGTTGAAAT | 29001-29020 | 1952 |
| GX01-R20 | TACCCTGAATGCTTGTCTGT | 30933-30952 |  |
| GX01-F21 | GACGATGATACCGCCCAAGT | 30760-30779 | 1753 |
| GX01-R21 | TGCTGAACAGGCACAAGATG | 32493-32512 |  |
| GX01-F22 | CCACAAATAAACTCCCAGAC | 32314-32333 | 1758 |
| GX01-R22 | GTCTCGACATCATGCCTCTG | 34052-34071 |  |
| GX01-F23 | GGATGCAAGGTGAAATCAGA | 33960-33979 | 1564 |
| GX01-R23 | TACAGGAAGGGTCGAGGGTA | 35504-35523 |  |
| GX01-F24 | ACAAAGTCCTCCTTCAAAAT | 35373-35392 | 1543 |
| GX01-R24 | CGTATGCAATGTCAAAAGGA | 36896-36915 |  |
| GX01-F25 | TCAAGATAAGCCGTTCCAAAG | 36687-36707 | 1829 |
| GX01-R25 | CGTACAGGTCTTTCCTATTTC | 38495-38515 |  |
| GX01-F26 | TACTCCTCAGCCTATCACAGA | 38307-38327 | 1814 |
| GX01-R26 | GGATACGGTAGTTGACTCCAT | 40100-40120 |  |
| GX01-F27 | CGCCCGTTTTTAATATCCGT | 39991-40010 | 1719 |
| GX01-R27 | TACCGTGTTCGTATTACAGC | 41690-41709 |  |
| GX01-F28 | CTCTAATAGTAACGCAATTGGA | 41600-41621 | 1488 |
| GX01-R28 | GAAGTGTGTTCTTTACTGGTAG | 43066-43087 |  |
| GX01-F29 | ATCACCCCTTCCTCTAAACC | 42795-42814 | 869 |
| GX01-R29 | ATGTAGGTATATATAGATGATG | 43642-43663 |  |

TABLE 3. Primers of quantitative real-time PCR assay

| Primers | Sequence (5’ to 3’) | Location of primers | Length, (bp) |
| --- | --- | --- | --- |
| qFAdV-52K-F | ATGGCGCAGATGGCTAAGG | 14180-14198 | 176 |
| qFAdV-52K-R | AGCGCCTGGGTCAAACCGA | 14337-14355 |  |


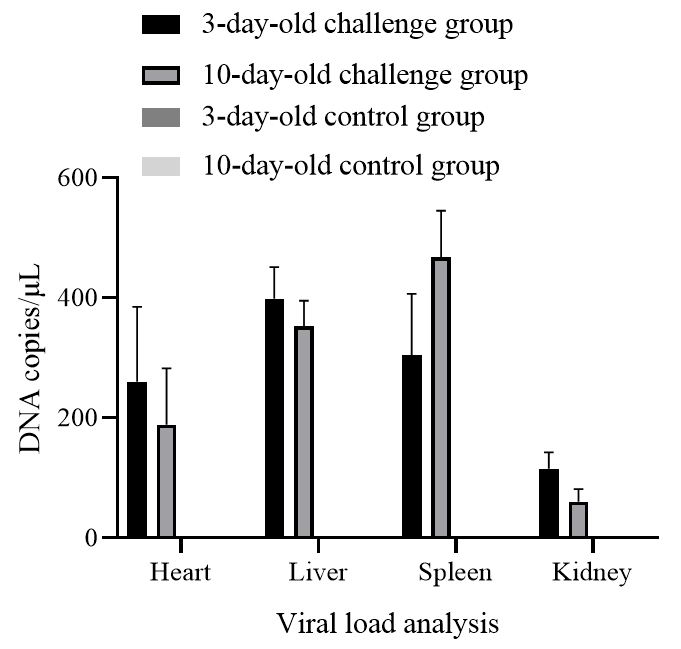


Supplementary Figure 1**｜**The viral load in various tissues of FAdV-2 strain GX01 infected chickens at 21 days post infection. n=3 chickens per group.
